# Probing plasmonic excitation mechanisms and far-field radiation of single-crystalline gold tapers with electrons

**DOI:** 10.1098/rsta.2019.0599

**Published:** 2020-10-26

**Authors:** Robin Lingstädt, Nahid Talebi, Surong Guo, Wilfried Sigle, Alfredo Campos, Mathieu Kociak, Martin Esmann, Simon F. Becker, Eiji Okunishi, Masaki Mukai, Christoph Lienau, Peter A. van Aken

**Affiliations:** 1 Max Planck Institute for Solid State Research, Stuttgart, Germany; 2 Institute of Experimental and Applied Physics, Christian Albrechts University, Kiel, Germany; 3 Facultad de Ciencias y Tecnología, Universidad Tecnológica de Panamá, Panama City, Panama; 4 Laboratoire de Physique des Solides, Université Paris Sud, Orsay, France; 5 Carl von Ossietzky University, Oldenburg, Germany; 6 CNRS Centre for Nanoscience and Nanotechnology (C2N), Université Paris-Saclay, Palaiseau, France; 7 JEOL Ltd., Tokyo, Japan

**Keywords:** gold taper, surface plasmon, cathodoluminescence, electron energy-loss spectroscopy, plasmonics

## Abstract

Conical metallic tapers represent an intriguing subclass of metallic nanostructures, as their plasmonic properties show interesting characteristics in strong correlation to their geometrical properties. This is important for possible applications such as in the field of scanning optical microscopy, as favourable plasmonic resonance behaviour can be tailored by optimizing structural parameters like surface roughness or opening angle. Here, we review our recent studies, where single-crystalline gold tapers were investigated experimentally by means of electron energy-loss and cathodoluminescence spectroscopy techniques inside electron microscopes, supported by theoretical finite-difference time-domain calculations. Through the study of tapers with various opening angles, the underlying resonance mechanisms are discussed.

This article is part of a discussion meeting issue ‘Dynamic *in situ* microscopy relating structure and function’.

## 1. Introduction

**Figure 1.**
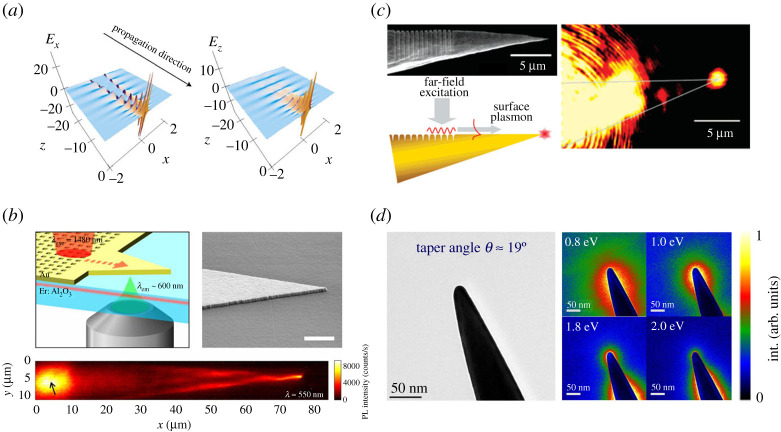
(*a*) Temporal snapshots of the excited local electric fields on the taper surface, plotted as a longitudinal cross section. Both normal ($E_{x}$, left panel) and longitudinal components ($E_{z}$, right panel) reveal an increased intensity at the apex (x=y=z=0). Adapted from ref. [5]. (*b*) Excitation of SPPs in a laterally tapered waveguide by coupling infrared light through a hole array. The SPP near field is mapped indirectly via upconversion luminescence spectroscopy (left panel). SEM image of the fabricated gold structure on an Erbium-doped sapphire substrate (right panel). Scale bar is 1 μm. Upconversion luminescence image of the structure (lower panel). The excitation position at the hole array is marked with a black arrow. Adapted from ref. [6]. (*c*) SEM image of a conical taper with a grating coupler to enable SPP excitation by far-field radiation (left panels). Optical microscope image showing radiation from the apex for the nonlocal excitation at the grating (right panel). Adapted from ref. [7]. (*d*) Zero-loss-peak-filtered bright-field TEM image of a taper with an opening angle of 19° (left panel). Energy-filtered TEM images at the depicted energies to spatially map plasmon resonances (right panel). Adapted from ref. [8]. (Online version in colour.)

In the field of nanophotonics, metallic nanostructures are well-known functional elements. In such structures, collective oscillations of free charge carriers, the so-called surface plasmons, can strongly interact with photons and therefore propagate and transport electromagnetic energy on the nanoscale, reaching confinements even substantially below the diffraction limit (figure 1*a*) [1–5].

In the case of surface plasmon [9–11], channel plasmon [12] and wedge plasmon [13,14] polaritons, long-range propagation has been found in planar geometries [6] (figure 1*b*).

One of the most established and widely used nanostructures are conically shaped metallic tapers, due to their practical application as waveguides or nanoantennas in scanning near-field optical microscopy (SNOM) [7,15–23] or as an ultrafast photoemission source in point-projection microscopes [24–33].

To investigate the underlying physical principles of these technologically important nanophotonic devices, several characterization techniques and numerical simulation methods have been employed to further understand excitation and propagation processes and the mechanism of focusing electromagnetic energy to the nanoscale (figure 1*c*) [7,19,32,34–38].

Being one of the pioneering methods to unravel the existence of plasmon oscillations at metal surfaces [39–43], electron energy-loss spectroscopy (EELS) in transmission electron microscopy (TEM) is able to detect both bright and dark plasmonic modes over a wide energy range [44–47]. Dark modes are defined via their diminishing coupling strength to a far-field plane-wave excitation. As spatial and energy resolution have been improved since then, EELS is still one of the best methods to map plasmon resonances of metallic nanostructures (figure 1*d*) [8,48–52]. Through extensive experimental and theoretical studies, it is well known that the electron energy-loss is directly related to the photonic local density of states (LDOS) projected along the electron trajectory [53] within an energy and momentum conservation criterion [54]. Interestingly, for mesoscopic rather than nanoscopic specimen dimensions, as will be discussed here, a dynamic exchange of momentum and energy is observed along the electron trajectory. While EELS probes the primary excitations, the radiative damping during the relaxation process can be measured by cathodoluminescence (CL) spectroscopy [48,55,56]. Especially, the combined study of an object by EELS and CL gives complementary insight into its physical properties [57–61]. However, numerical calculations are essential to interpret experimental results and to understand the physics behind them. For instance, it is well known that the fundamental transverse magnetic (TM) surface plasmon polariton (SPP) mode with vanishing angular momentum m=0 is able to propagate along the taper shaft and concentrate the carried electromagnetic energy at the apex, while its radially symmetric electric field is evanescently bound to the surface [17,35,62]. This coupling to localized plasmons at the tip is termed as adiabatic nanofocusing (figure 1*a*) [5,7,36]. By contrast, other plasmon modes with higher angular momentum order |m|>0 do only propagate above certain critical radii (i.e. the local radius of the cross section normal to the taper axis) where they radiate off the taper and couple to the far-field [34,35,38,63]. For realistic taper geometries with limited radius of curvature at the apex, both reflection and radiation of the m=0 mode have been observed [24,32]. In general, the behaviour of the different plasmonic modes, sustained by metallic tapers, is strongly affected by varying parameters like apex curvature, opening angle or surface quality, which was addressed by recent studies [32,34,64–66].

In this review article, an overview will be given about recent developments and results in the field of plasmonic nanostructures. Three-dimensional single-crystalline gold tapers with different opening angles were investigated experimentally by both EELS and CL, and supported by numerical calculations. The proposed theoretical model reveals a dynamic interplay of the plasmonic modes with the exciting swift electron, which results in the observed interference pattern in the EELS signal. In general, two coexisting mechanisms are identified, that are more or less dominant, depending on the taper geometry. While the reflection of plasmonic modes from the apex plays the major role for tapers with small opening angles (below 10°), the phase-matching between the electric fields of plasmonic modes with higher-order angular momentum and the passing electron becomes dominant for large opening angles (above 20°), due to the longer interaction time.

## 2. Results

Single-crystalline gold tapers [36,67] with a particularly smooth surface were used for the experiments in order to minimize scattering losses and localization of SPPs [68]. EELS measurements were performed at an acceleration voltage of 200 kV with the ZEISS SESAM microscope, a dedicated instrument for low-loss experiments with high-energy resolution [69]. As the measured EELS intensity represents the probability of the relativistic electron to excite surface plasmons along its trajectory, this technique is ideally suited to measure the photonic LDOS for broadband excitations in the energy range from meV to hundreds of eV. Combined with the spatial information provided by energy-filtered TEM or by scanning the electron probe over an area of interest to obtain a spectrum image, mapped intensity variations reveal resonance effects and give insight into the strong dependence of plasmonic modes on taper geometries. For gold, however, this spatial information is limited to energies below 2 eV, where interband absorptions set in and result in a homogeneous EELS intensity distribution [70,71]. Due to experimental constraints of the used electron microscope, the energy resolution is limited to slightly below 100 meV. However, with state-of-the-art instruments values below 10 meV have become accessible, which corresponds to the energy range of THz spectroscopy.

**Figure 2.**
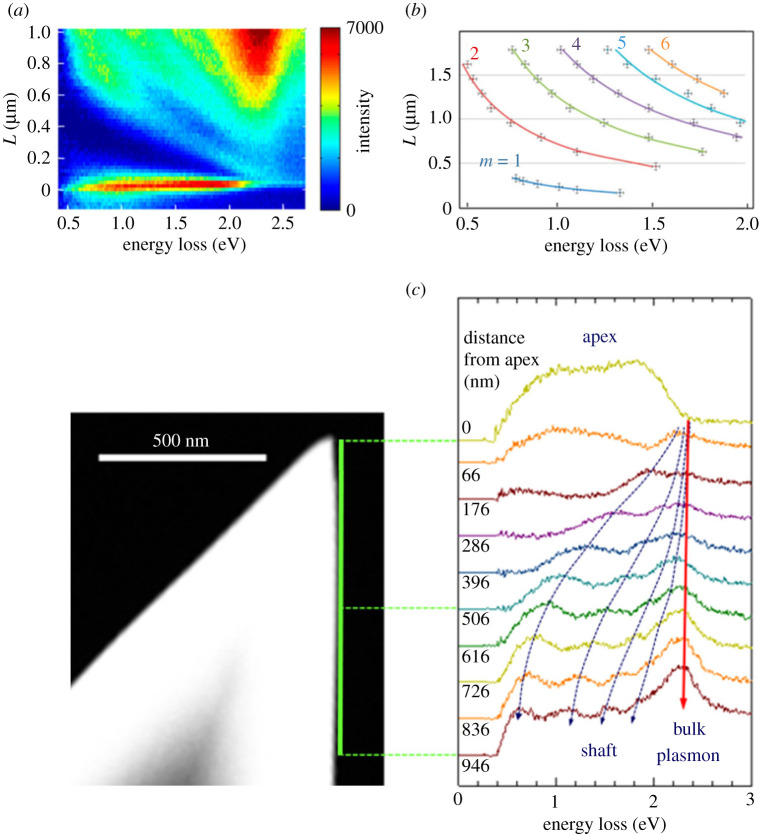
(*a*) Local EELS spectra of a conical gold taper with an opening angle of 49°. The energy-distance map shows the EELS intensity over the scanning distance *L*. A very intense broadband signal is detected at the taper apex and higher-order angular momentum eigenmodes become apparent. (*b*) Taper eigenmodes with higher-order angular momentum numbers m=1,…,6 are identified by least-squares fits of hyperbolic functions to the maxima in the energy-loss spectra, and traced over the scanning distance *L*. Adapted from ref. [34]. (*c*) Background-subtracted energy-loss spectra, extracted at variable distances from the apex along the shaft of a taper with an opening angle of 45°, as depicted in the dark-field TEM image on the left. The spectra are shifted vertically for clarity. The spectra show a clear broadband feature at the apex and a peak at the bulk plasmon energy around 2.3 eV throughout the line scan, due to the interaction of the electron with the gold material. In addition, an increasing number of intensity maxima is observed in the energy-loss spectra at larger distances to the apex, that also shift towards lower energies. (Online version in colour.)

As indicated by the green line next to the dark-field image of a taper with an opening angle of 45° (figure 2*c*), the electron probe is scanned along the taper shaft for the experiments. At the apex, a very intense signal is detected, which is associated with highly confined localized surface plasmons. Its spectral intensity is almost constant over a wide energy range (figure 2*a*,*c*), in contrast to results of earlier work on metallic nanoparticles and conical tapers, where this broadband characteristic has not been observed [72]. Moving the electron probe further away from the apex, distinct maxima are observed in the spectra below 2 eV that shift towards lower energies. These resonances are related to higher-order angular momentum taper eigenmodes, as will be discussed below. Other experiments, carried out by SNOM within the strong-interaction regime, revealed spectral features that shift towards higher energies instead. These modes are responsible for the multiple scatterings between the specimen and the SNOM probe and hint at the coexistence of two different modes with orthonormal polarization properties [23,73].

Detecting the energy-loss spectrum continuously over a certain scan distance *L* along the taper shaft results in an energy-distance map (figure 2*a*). The individual modes can be identified by tracing maxima in the spectra over *L* and fitting the data with hyperbolic functions $E=E_{0}+\kappa /L$, with $E_{0}$ and κ being constants (figure 2*b*). Moreover, the fitting parameters were found to linearly depend on the mode number *m*, resulting in the empirical relation

$$E=(0.105\;m-0.098)\text{eV}+\frac{\left(0.139\;m-0.014\right)}{R}\text{μ}m\cdot \text{eV},$$

with R=L⋅sin⁡(α/2) as the local radius at a certain distance from the apex.

Numerical calculations revealed that the observed resonances can be attributed to a phase-matching mechanism between the electric fields of taper modes and the exciting electron and will be discussed in the following.

Aiming for a more fundamental level of understanding the physical principles behind these experimental observations, conical structures have been extensively studied theoretically in the past. Although rather complex mathematical methods and novel approximation schemes have been applied, the determination of explicit solutions is still a matter of ongoing research [74–76]. Following the comparably intuitive approach of viewing the taper as a combination of infinitely thin slices of metallic fibres with continuously varying radii, the near-field properties of a conical structure can be effectively modelled by a superposition of fibre eigenmodes [34]. After solving the Helmholtz equation in cylindrical coordinates, they can be characterized by the complex wavenumber $k_{z}$ along the fibre axis and the azimuthal angular momentum number m=0,±1,±2,… [77,78]. Interestingly, higher-order modes can only propagate evanescently bound to the metallic surface above a certain mode-dependent critical local radius, where they radiate off the taper and couple to the continuum of photonic modes. By contrast, it is only the rotationally symmetric m=0 mode that is not restricted in this regard. This lowest-order angular momentum mode is therefore able to transport electromagnetic energy to the apex and focus it adiabatically [5,79–81].

**Figure 3.**
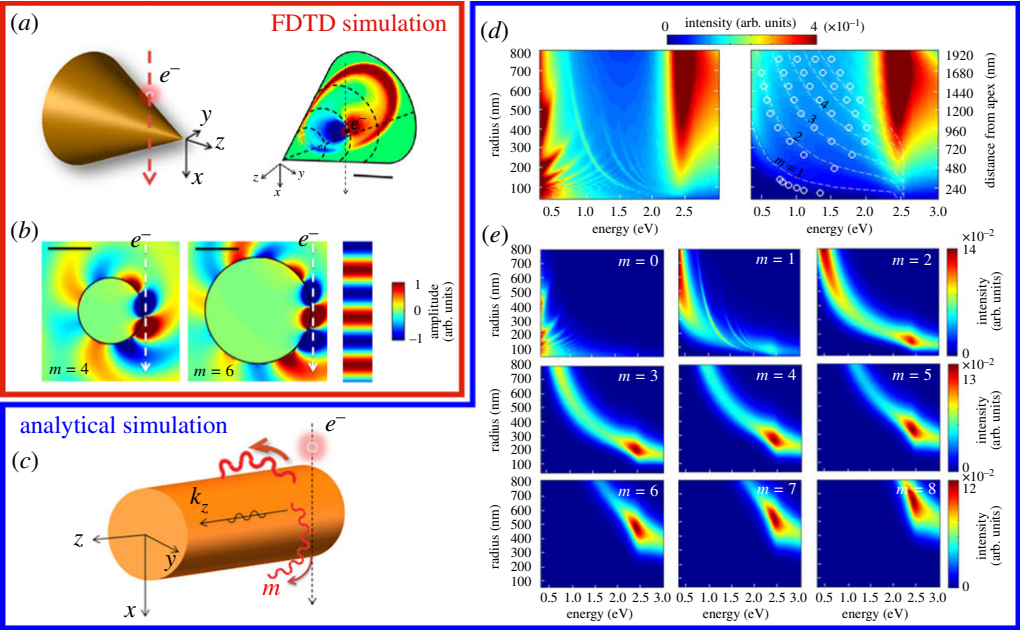
(*a*) Three-dimensional sketch (left panel) and dynamic simulation for a taper with an opening angle of 45° (right panel), showing the $E_{z}$-field on its surface, while an electron passes the structure 900 nm away from the apex. The colour code maps the field distribution 1 fs before the electron reaches its closest distance of 1 nm from the surface. (*b*) Scattered electric field component $E_{z}$, calculated for an energy-loss of 1.6 eV, induced by an electron that passes the taper at the local radii R=383 nm (left panel) and R=612 nm (centre panel). The plane-wave component of the electric field, corresponding to the moving electron, is displayed on the right. Scale bars are 500 nm. (*c*) A fast electron passes an infinitely long metallic fibre at a close distance. Due to the interaction, evanescent SPP modes are excited that propagate along the fibre $\left(k_{z}\right)$, as well as radiative modes with different angular momentum numbers *m*. (*d*) Energy-loss spectra simulated for different fibre radii including (left panel) and excluding (right panel) modes with $k_{z}>k_{0}$ for an electron that passes at a distance of 1 nm from the surface. Experimentally determined values (circles), measured along a taper with an opening angle of 49°, resemble the theoretically predicted EELS maxima very well (dashed lines). (*e*) Simulated contribution of the individual modes with angular momentum numbers m=0,…,8. Adapted from ref. [34]. (Online version in colour.)

The conducted experiments were evaluated theoretically by finite-difference time-domain (FDTD) simulations. The ability to calculate electric field distributions and their evolution with high temporal resolution gives insight into the very fast interaction processes of the passing electron with the investigated structure. Of course, it is essential to model the entire experimental set-up very accurately, including the three-dimensional taper itself and the relativistic electron source [55,82]. The results of a dynamic simulation for a gold taper with an opening angle of 45° in the aforementioned local radius approximation are shown in figure 3*a*,*b*. The structure is excited by a fast-moving electron, which passes the surface at a minimum distance of 1 nm. Its speed is set to roughly 70% of the speed of light, which corresponds to an acceleration voltage of 200 kV in an electron microscope. A detailed analysis of the temporal and spatial evolution of the fields revealed that most of the transferred energy is radiated in the form of an ultrashort light pulse, whereas only a minor amount couples to evanescent surface modes. Although initially a wave packet with a broad energy distribution and wide range of mode numbers *m* and wavenumbers $k_{z}$ is launched, only modes up to a certain mode order *m* can propagate towards the apex due to the limiting local radius at the impact position. Energy-loss spectra were simulated for infinitely long fibres with varying radii (figure 3*d*). The calculated results clearly reveal a resonance characteristic in good agreement with the experimental findings (figure 2*b*). The observed hyperbolic dispersion of energy-loss maxima depending on the local radius were analysed carefully with regard to the underlying model of infinitely long fibres [34]. The development of an analytical description, by writing the energy-loss probability as a sum over the mode number with subsequent Fourier analysis in both wavenumber and frequency domain, revealed two different mechanisms as reasons for EELS resonances. Firstly, absorption maxima are expected, whenever the spatial profile of the scattered field of an excited fibre matches closely with the field of one of its eigenmodes that propagate along the taper (or fibre) axis [77,78]. Secondly, by evaluating overlap integrals along the electron trajectory, within the fully expanded EELS probability, it was shown that the spatial phase pattern of the passing electron can resemble one of the rotating eigenmodes with higher angular momentum orders for certain energies. Whenever this phase-matching occurs, energy is resonantly transferred from the electron to the structure and an absorption maximum is observed in the energy-loss spectrum. This case is exemplarily shown for an assumed frequency ω=1.6 eV/ℏ, for which the electron field oscillates along its trajectory with a wavelength of 539 nm (figure 3*b*). Fourier transformations of the FDTD simulations revealed a strongly scattered field for that particular frequency only, if the electron passes the taper at specific local radii. In addition, the field distributions closely resemble the field pattern of the fibre eigenmodes with m=4 and m=6, respectively. A rather rough approximation can be made by evaluating the aforementioned overlap integral only over a reduced interaction length, as the interaction is the strongest close to the surface. Phase-matching resonances are observed whenever the phase of the electron field matches with the phase of a fibre mode field over the interaction length. The obtained hyperbolic dependence of resonance energies on local radii $\hbar \omega \approx (m\pm 1)\hbar v_{\text{el}}/R$, where $v_{\text{el}}$ is the electron velocity, resembles the empirically determined dispersion relation qualitatively quite well.

In addition to the EELS experiments, where the absorption probability is dispersed into energy spectra, the theoretical model allows for further analysis regarding the decomposition versus longitudinal and angular momenta along and around the fibre axis, respectively. The calculations show that for comparably thin fibres (R=50 nm)  the rotationally symmetric m=0 eigenmode most dominantly contributes to the EELS signal and phase-matching resonances are not relevant. For larger radii, however, the situation is completely different. Due to the increased interaction length along the electron trajectory, the EELS signal is most strongly influenced by the phase-matching mechanism rather than the excitation of fibre eigenmodes. As it was shown for the fibre radius R=400 nm, resonance energies, surprisingly, correspond to highly radiative modes with $k_{z}<k_{0}$, where $k_{0}=E/\hbar c$ is the wavenumber of light in vacuum. For the interpretation of EELS data of conical tapers and finite structures in general, with dimensions that exceed multiples of the electron field oscillation wavelength along its trajectory, the phase-matching mechanism definitely has to be taken into account. It should be noted, however, that the results depend on the electron velocity and that standing wave patterns may arise due to the reflection of the propagating fundamental mode at the apex, especially for tapers with narrow opening angles [32].

**Figure 4.**
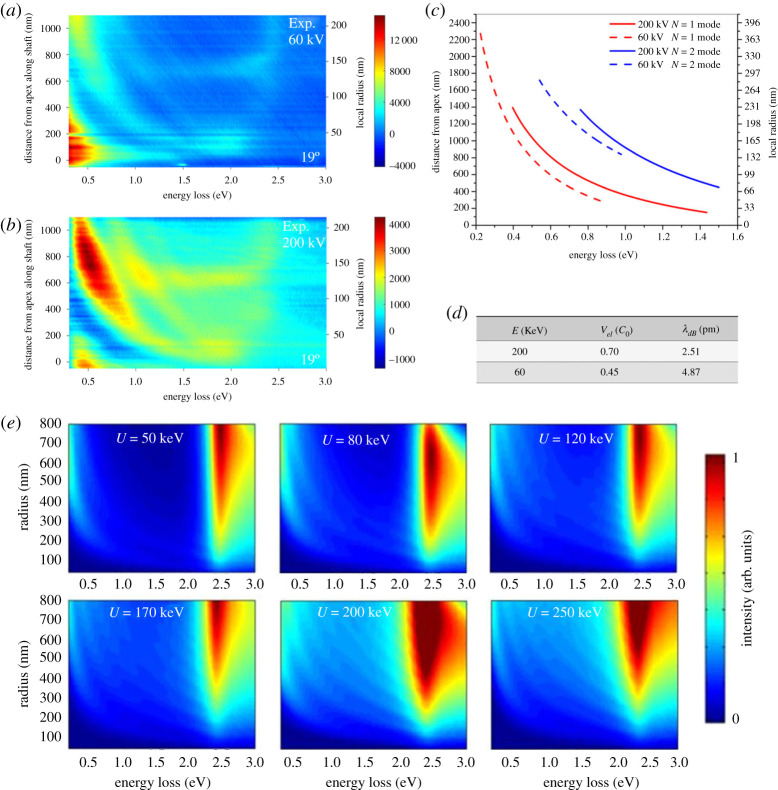
(*a*) Experimental EELS line scans along a taper with an opening angle of 19° for acceleration voltages of 200 kV (*a*) and 60 kV (*b*). The dispersions of extracted maxima (*c*) show a clear redshift for 60 keV electrons (dashed lines) for the first two mode orders (lower red curve and upper blue curve) compared with 200 keV electrons (solid lines). The associated relativistic electron velocities and correlated de Broglie wavelengths are shown in (*d*), where $c_{0}$ is the speed of light in vacuum. (*e*) Simulated EELS line scans for the depicted electron energies reveal a blue shift of the resonance dispersions with increasing electron velocity. (Online version in colour.)

The aforementioned hyperbolic equation for phase-matching resonances $\hbar \omega \approx (m\pm 1)\hbar v_{\text{el}}/R$ contains a scaling factor that in turn depends on the relativistic speed $v_{\text{el}}$ of the swift electron. Lowering the acceleration voltage of the electron microscope therefore implies a redshift of the resonance dispersions. By contrast, effects caused by the reflection mechanism remain constant, as they explicitly depend on the taper geometry. Spectral EELS line scans have been performed, using a monochromated JEOL electron microscope, along a gold taper with an opening angle of 19° for both 200 and 60 keV electrons (figure 4*a*,*b*,*d*). Tracing the maxima in the experimental spectral dispersions for the first two mode orders clearly reveals a redshift for the lower acceleration voltage (figure 4*c*), which was predicted by numerical calculations for various acceleration voltages (figure 4*e*). These measurements therefore directly prove the existence of the phase-matching mechanism in plasmonic gold tapers.

**Figure 5.**
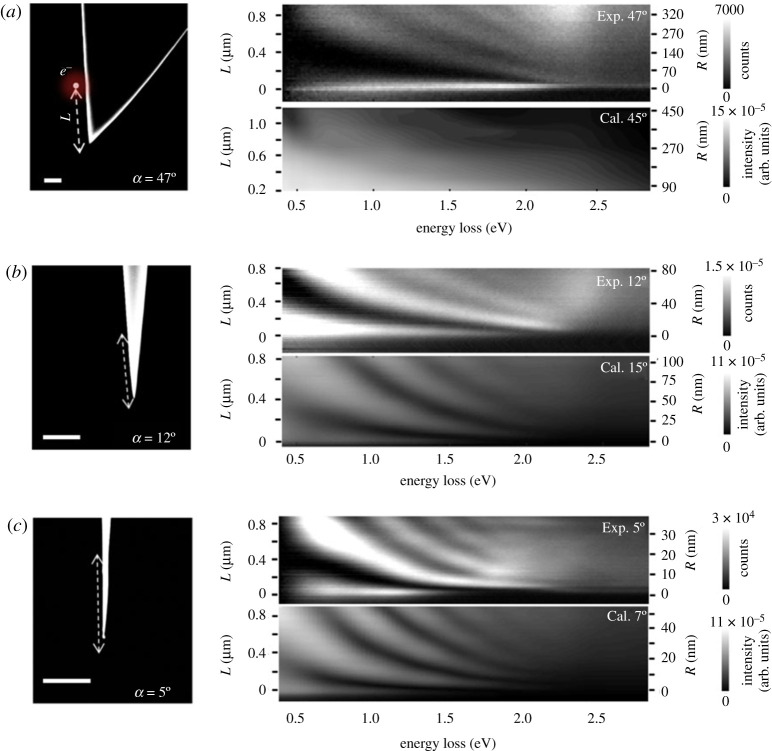
Experimental and calculated energy-loss dispersions, measured for tapers with opening angles of 47° (*a*), 12° (*b*) and 5° (*c*) while scanning the electron probe in close distance along the taper shaft starting at the apex (L=0). Experimental spectra were background subtracted by applying a power-law fit. Scale bars are 500 nm. Adapted from ref. [83]. (Online version in colour.)

**Figure 6.**
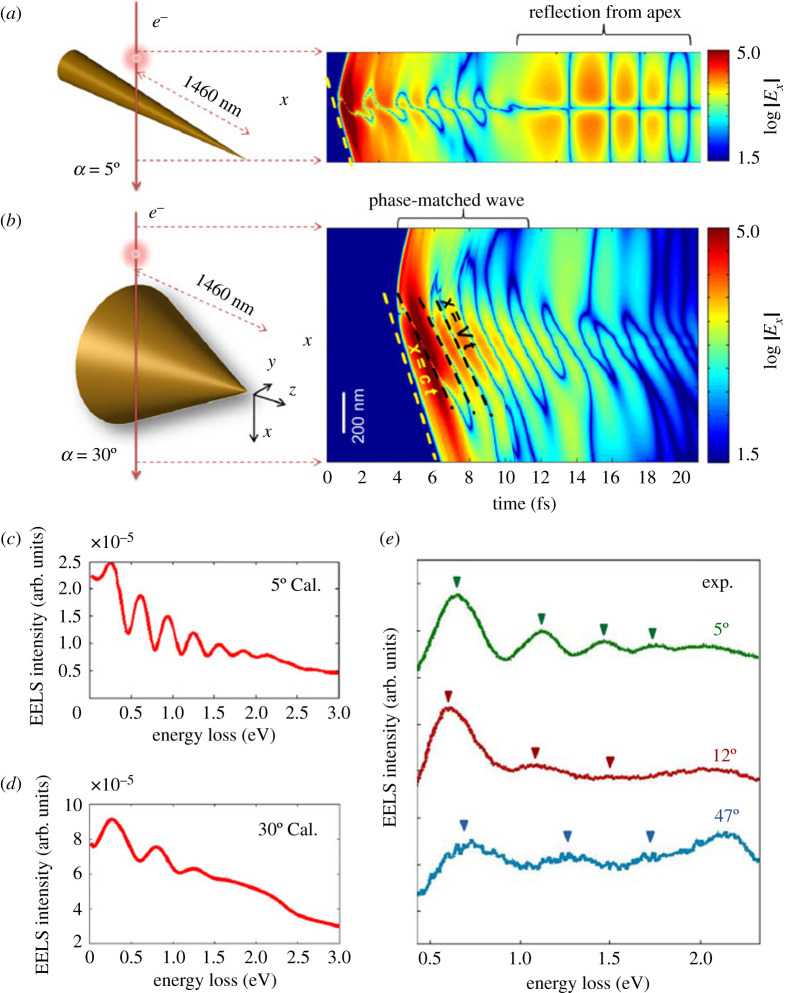
FDTD simulations for gold tapers with 5° (*a*) and 30° (*b*) opening angles that are excited by a fast electron at a position 1460 nm away from the apex. The scattered electric field, projected along the electron trajectory $|E_{x}|$, is plotted against the x-position and time on a logarithmic scale. Plasmon oscillations lead to the emission of electric fields that propagate away from the structure at the speed of light (yellow dashed line). In addition, higher-order angular momentum modes result in a complex interference pattern. A clear time-delayed signature of the fundamental mode, being reflected at the apex, is observed for the taper with 5° opening angle (*a*). For the taper with large opening angle (*b*), also modes with higher-order angular momentum (m=1 and m=2) can propagate along the shaft. They are evanescently bound to the surface as a SPP wave packet, with spatio-temporal dynamics that depend on the electron velocity (black dashed line). The calculated energy-loss spectrum for the narrow taper (*c*) is dominated by the reflection mechanism, whereas phase-matching plays the major role for large opening angles (*d*). (*e*) Experimental spectra for gold tapers with varying opening angles, acquired at a position 800 nm away from the apex. Adapted from ref. [83]. (Online version in colour.)

As mentioned earlier, analytical models for conical metal tapers confirmed the coexistence of the two mentioned mechanisms and their effect on the EELS signal [38]. To investigate their origin further, single-crystalline gold tapers with various opening angles ranging from 5° to 47° were studied both experimentally by EELS and theoretically by FDTD simulations (figure 5) [83]. The simulated results for the two extreme geometries 5° and 30° are shown in figure 6*a,b*, respectively. The structures were excited by relativistic electrons with a kinetic energy of 200 keV that pass the tapers at a distance of L=1460 nm from the apex. In the chosen orientation of the coordinate system, the x-component of the scattered electric field exclusively contributes to the EELS signal, as it is oriented parallel to the electron trajectory. Its value is plotted on a logarithmic scale in dependence of the x-position along the electron trajectory and the evolved time. The time dependence of the reemitted fields of both structures shows similarities on the one hand, but also fundamentally different characteristics. In both cases, plasmonic modes with higher-order angular momentum are excited that result in the formation of a complex interference pattern. In addition, they radiate off the taper and launch electromagnetic fields that propagate through free space at the speed of light (yellow dashed lines in figure 6*a,b*). For the narrow taper with an opening angle of 5°, only the fundamental mode with m=0 is evanescently bound to the surface and can propagate to the apex, where it is partly reflected and results in an evanescent field burst, that arrives at the excitation location with a time delay of approximately 11 fs (figure 6*a*) [84]. At the position where the electron comes closest to the surface, both longitudinal and radial components of the propagating mode vanish when projected onto the electron trajectory, resulting in a centred line of zero intensity. For the taper with 30° opening angle, also higher-order modes are evanescently bound and can propagate away from the impact position. As a result, their propagation occurs in the form of a wave packet. Due to the increased interaction length with the electron along its trajectory for larger local radii, the resulting interference pattern is inclined by a different angle (black dashed line in figure 6*b*), which in turn depends on the electron velocity. The two different mechanisms ‘reflection’ and ‘phase-matching’ that dominate the plasmonic response of tapers with narrow and wide opening angle, respectively, also result in a different signature in the spectral domain. While the time-delayed oscillations cause pronounced peaks in the energy-loss spectrum of the 5° taper with decreasing fringe distances for higher energies (figure 6*c*) [38], the modulation in the spectrum of the 30° taper arises mainly from the excitation of higher-order angular momentum modes, which couple to the far-field before reaching the apex (figure 6*d*). The back-reflected signal of the fundamental mode is comparably weak, so that the phase-matching mechanism remains the dominant contribution to the EELS signal.

Experiments revealed a similar trend in the spectra of gold tapers with various opening angles, which have been acquired at a distance of L=800 nm from the apex (figure 6*e*). Interestingly, the modulation contrast seems to decrease for tapers with intermediate opening angles like 12°, which might be the result of both contributions interfering in the same energy range. Slight differences between experimental results and theoretical calculations might be attributed to deviations of real tapers compared with ideal structures like the shape of the apex and cone or its surface roughness.

The gradual transition from phase-matching-dominated resonances to a reflection-based origin can be illustrated in a simplified picture, as tapered structures more and more resemble a semi-infinite whisker by decreasing the opening angle. For a gold whisker with a radius R=50 nm, only the fundamental m=0 order mode can propagate. Comparing the total scattered field projected along the electron trajectory $|E_{x}|$ at a distance of L=1460 nm from the end, the purely reflection-based response of the whisker is gradually suppressed by the phase-matching mechanism for tapers with increasing opening angle, which finally evolves to be the dominant feature for the taper with 50° opening angle.

Energy-loss spectra were experimentally acquired along a gold whisker [85] with a radius of 50 nm and a gold taper with an opening angle of 47° over a variable distance to the end and apex, respectively. The resulting energy-distance maps were evaluated by tracing maxima in the EELS signal over the scan distance. Although the dispersions show similarities, their intrinsic origin and physical interpretation differ significantly. The experimental data were fitted with hyperbolic functions of the forms $E=\kappa_{L}/(L+L_{0})$ and $E=\kappa_{R}/(R+R_{0})$, where R=L⋅sin⁡(α/2) is the local radius of the taper with opening angle α at the distance *L* to the apex. For both structures, κ is a constant that linearly depends on the mode order. The constant $L_{0}$ is interpreted as a virtual length increase of the whiskers end of about 200 nm [86,87], whereas $R_{0}$ extends the interaction length of the SPP field with the fast electron.

**Figure 7.**
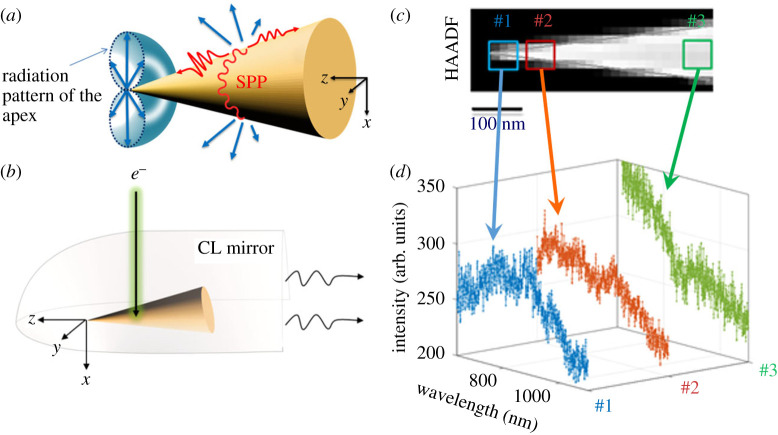
(*a*) Sketch of a plasmonic gold taper with sustained SPPs (red colour) that propagate in the forward, backward and azimuthal directions. Their decay leads to far-field radiation (straight arrows in blue colour) from the surface and the apex [36] that is collected by a parabolic mirror (*b*) and focused onto a spectrometer. Spectra (*d*) can be extracted for different excitation positions within the scanned area and are marked in the High-Angle Annular Dark-Field (HAADF) image (*c*). Adapted from ref. [66]. (Online version in colour.)

In addition to EELS experiments, where energy-loss probabilities due to local excitation processes are probed, the aforementioned far-field radiation of plasmonic tapers was spectroscopically analysed by CL measurements for two tapers with opening angles of 13° and 47° [66]. The experiments were conducted with a scanning transmission electron microscope Vacuum Generator HB501, which is equipped with a cold-field emission electron gun and operated at an acceleration voltage of 100 kV. For spatial mapping, the few-nanometre-sized electron probe with a comparably large beam current of around 1 nA is raster scanned over the area of interest. Far-field radiation that is scattered off the entire microscopic structure (figure 7*a*) is analysed with the Attolight Mönch STEM-CL set-up, consisting of a parabolic mirror (figure 7*b*) that focuses the emitted light to a spectrometer through an optical fibre bundle. The acquired emission spectra for each spatial pixel build up a multi-dimensional data cube that can be evaluated by different forms of representation, such as spatially resolved intensity variations of selected wavelengths or extracted spectra from individual positions (figure 7*d*). Highest CL intensities were measured for probe positions at certain distances to the apex, which result from a more efficient plasmonic excitation of the structure.

**Figure 8.**
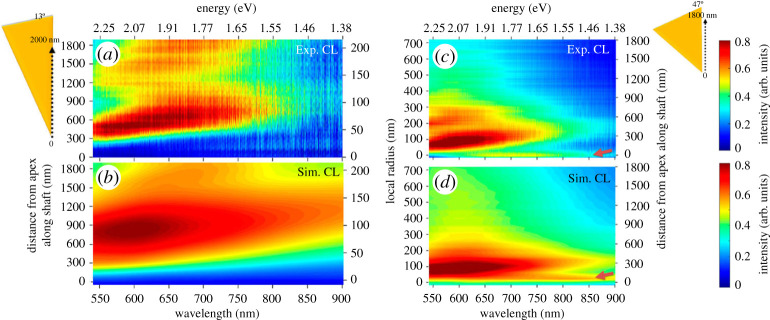
Experimental (*a,c*) and simulated (*b,d*) CL spectra as a function of the probe distance to the apex for tapers with an opening angle of 13° (left) and 47° (right). The corresponding local radii are shown on the second ordinate of the diagrams. The scale for the spectral domain is indexed for both wavelength and energy. Adapted from ref. [66]. (Online version in colour.)

This observation is confirmed by spectral line scans of the electron probe along the shaft of both tapers (figure 8). For the 13° taper (figure 8*a*), strong CL intensities arise for wavelengths in the range between 500 and 700 nm, when the taper is excited at a distance of roughly 300 nm from the apex. Furthermore, a redshift is observed for larger apex distances up to 800 nm, beyond which the intensity decreases due to damping effects. In addition to the experimental results, CL spectra were simulated based on numerical solutions to Maxwell's equations for the specific taper geometry (figure 8*b*). The good qualitative agreement implies that the observed CL radiation pattern mainly originates from the coherent far-field radiation of transverse electric and TM modes. Interestingly, although the local field enhancement is huge at the very apex [88,89], no significant radiation is observed for this particular excitation position. However, as only the m=0 order mode can propagate for local radii below 50 nm, energy is being carried away from the apex through backpropagation. This mechanism can be seen as a reciprocal process to the principle of adiabatic nanofocusing. In the case of the 47° taper, a similar radiation pattern is observed above a certain critical local radius for both experimental and simulated results (figure 8*c*,*d*). In addition, a broad spectral feature between 600 and 800 nm arises for an excitation position at the very apex. For the drastically changing local radius, the adiabaticity condition fails and the excited mode radiates to the far-field. For both taper geometries, the major radiation is observed for excitation positions above a certain distance from the apex and originates from the decay of higher-order angular momentum modes with |m|>0 [7].

## 3. Summary

Conical metallic nanostructures in the form of single-crystalline gold tapers were investigated experimentally by means of electron energy-loss and CL spectroscopy inside TEM. Supported by analytical and numerical FDTD simulations, it could be shown that resonances in the EELS signal originate from taper eigenmodes with higher-order angular momenta that interact with the exciting electron via a phase-matching mechanism. This effect was proven by measurements at different acceleration voltages, as it depends on the electron velocity. Modelling conical tapers in a local radius approach by infinitely long fibres along with measurements of tapers with various opening angles revealed that the back reflection of the fundamental mode at the apex becomes the dominant effect for narrow opening angles. The plasmonic excitation is most efficient at a certain distance to the apex and adiabatic nanofocusing fails for large opening angles, as it could be shown by spatially resolved far-field measurements.

The results presented in this review and also in related work [32,38] show that a combination of EELS, CL and three-dimensional simulations of Maxwell's equations can provide valuable insight into the optical properties of sharp metallic tapers as used, for instance, in scattering-type SNOM or ultrafast electron emission. Specifically, the electron microscopy experiments provide measurements of the full electromagnetic and radiative LDOS in a broad spectral range and with high spatial resolution, difficult to obtain by all-optical means. As such, EELS and CL will certainly be helpful for studying wider classes of SNOM probes or other types of point probe emitters. Such studies will greatly benefit from a detailed comparison of the experimental results to careful simulations of Maxwell's equations for the complex, three-dimensional objects under study. Together, such experiment–theory collaborations are likely to provide crucial new insights into the nanoscopic optical properties of various types of SNOM probes, their near-field coupling to the nanostructured surface under investigation and, more generally, different classes of metallic, dielectric or hybrid nanostructures. The promising results presented in this review encourage more work in this direction.

## Supplementary Material

Figures Licenses

## Supplementary Material

Ref 5 license author

## Data accessibility

This article does not contain any additional data.
