# Supplementary figures and images for: Probing plasmonic excitation mechanisms and far-field radiation of single-crystalline gold tapers with electrons
Source: Philos Trans A Math Phys Eng Sci. 2020 Oct 26;378(2186):20190599. doi: 10.1098/rsta.2019.0599 (PMC7661279; doi:10.1098/rsta.2019.0599)

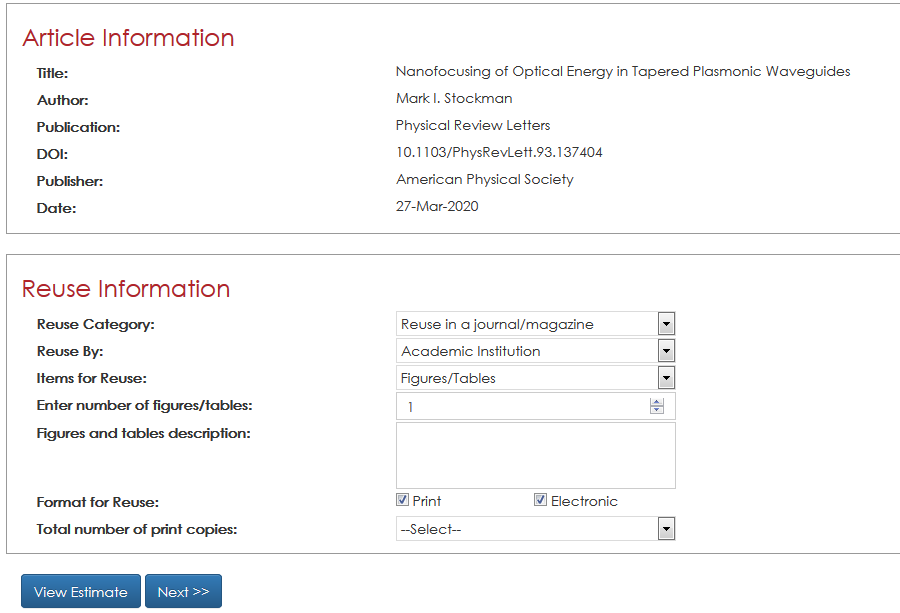

Supplement: Figures Licenses [file rsta20190599supp1.zip › Ref_5_license_a.PNG]

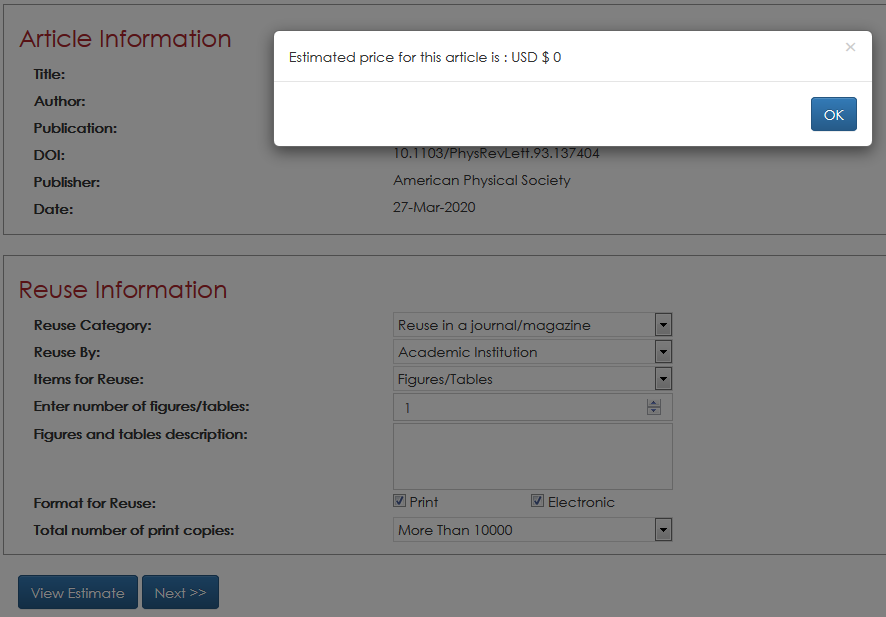

Supplement: Figures Licenses [file rsta20190599supp1.zip › Ref_5_license_b.PNG]

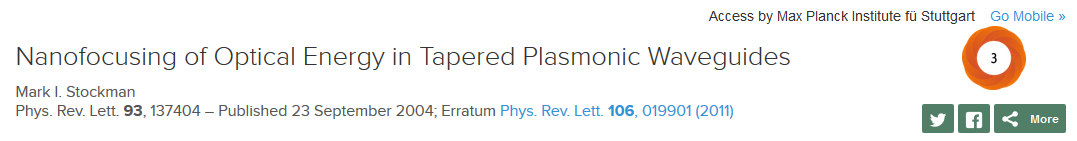

Supplement: Figures Licenses [file rsta20190599supp1.zip › Ref_5_title.PNG]

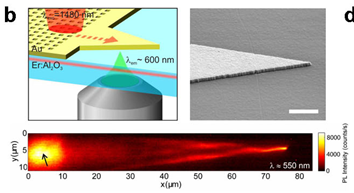

Supplement: Figures Licenses [file rsta20190599supp1.zip › Ref_12.PNG]

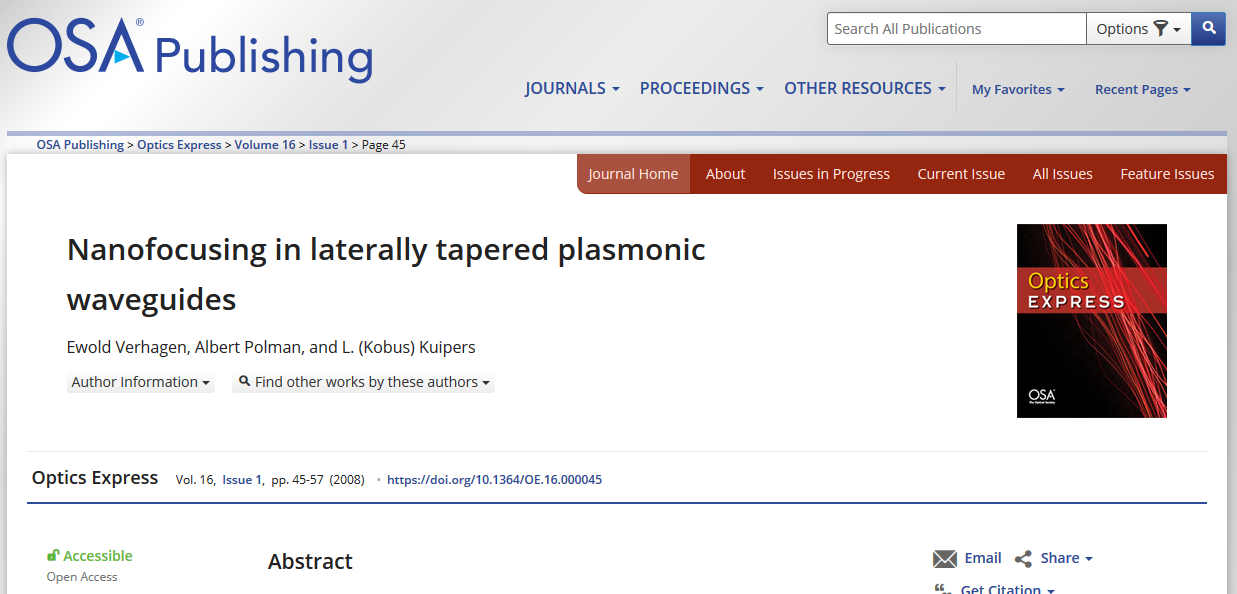

Supplement: Figures Licenses [file rsta20190599supp1.zip › Ref_12_license_a.PNG]

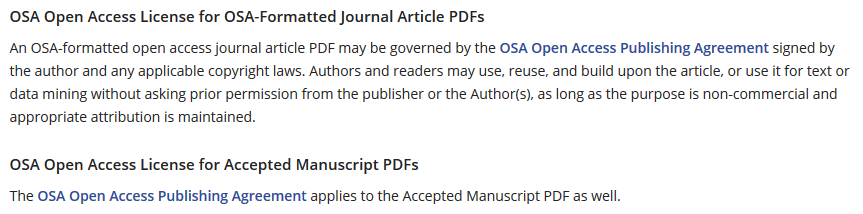

Supplement: Figures Licenses [file rsta20190599supp1.zip › Ref_12_license_b.PNG]

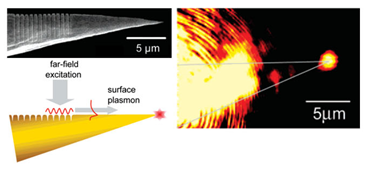

Supplement: Figures Licenses [file rsta20190599supp1.zip › Ref_15.PNG]

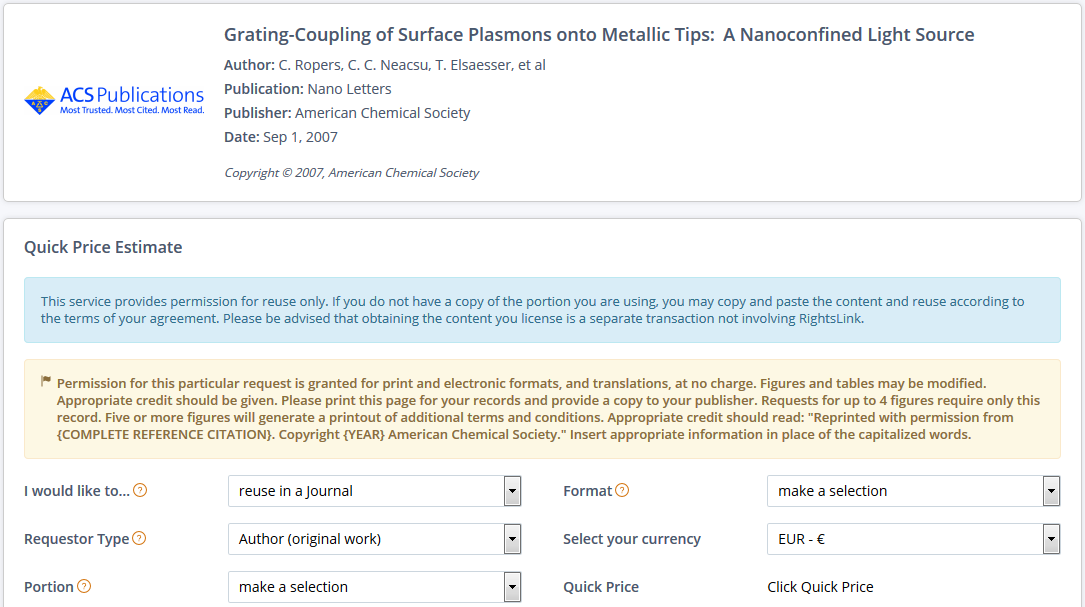

Supplement: Figures Licenses [file rsta20190599supp1.zip › Ref_15_license_a.PNG]

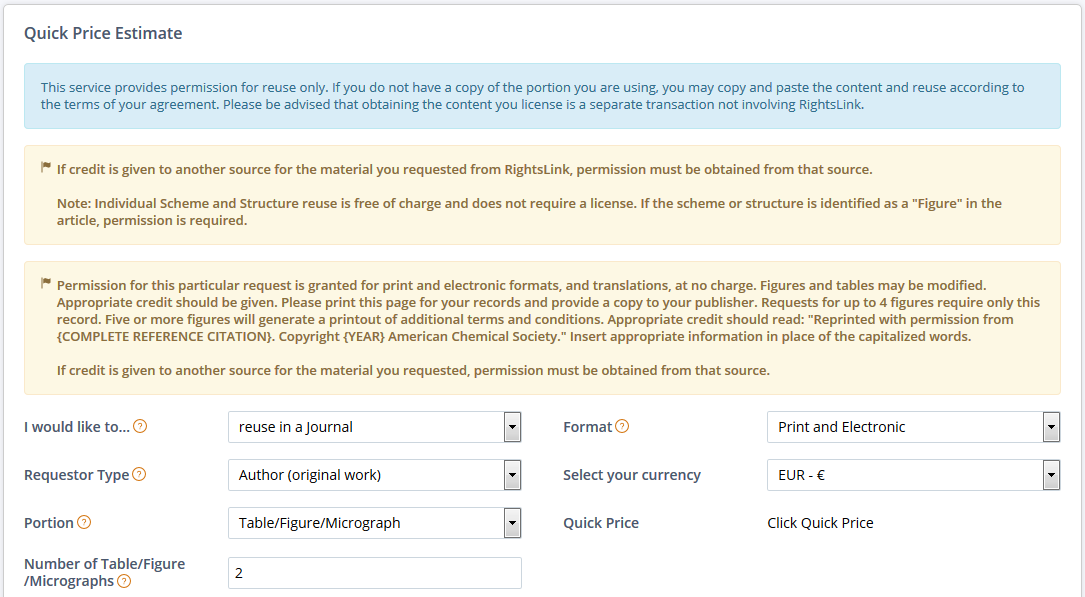

Supplement: Figures Licenses [file rsta20190599supp1.zip › Ref_15_license_b.PNG]

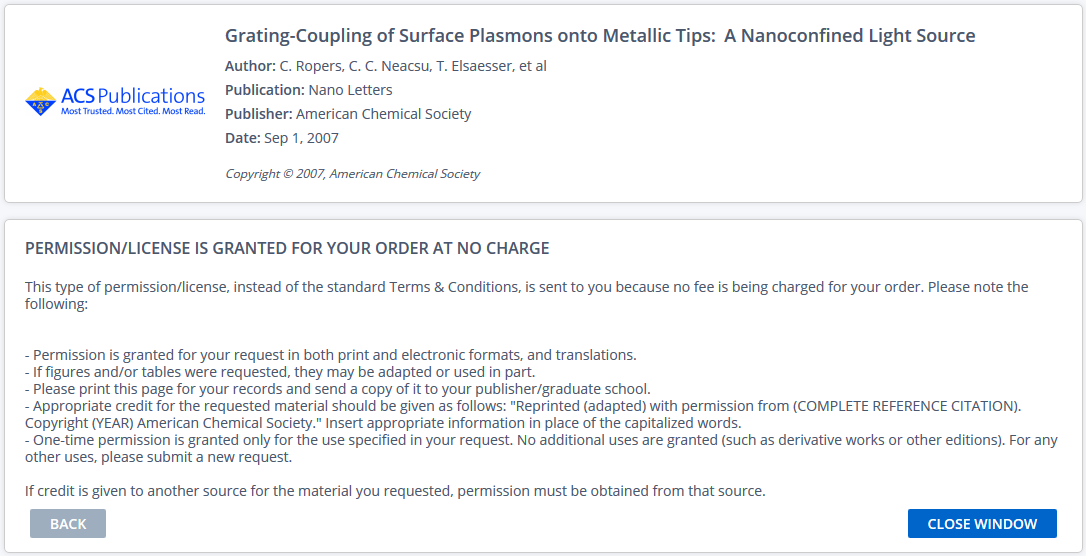

Supplement: Figures Licenses [file rsta20190599supp1.zip › Ref_15_license_c.PNG]

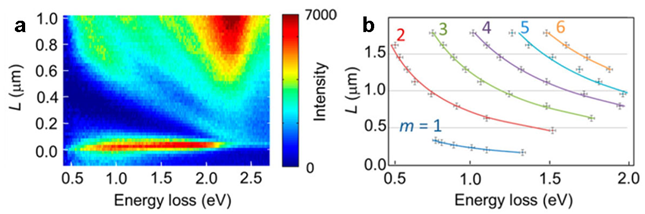

Supplement: Figures Licenses [file rsta20190599supp1.zip › Ref_33_a.PNG]

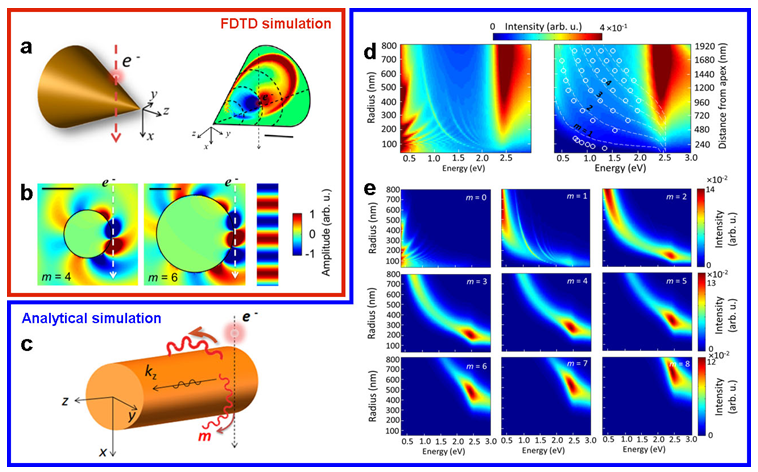

Supplement: Figures Licenses [file rsta20190599supp1.zip › Ref_33_b.PNG]

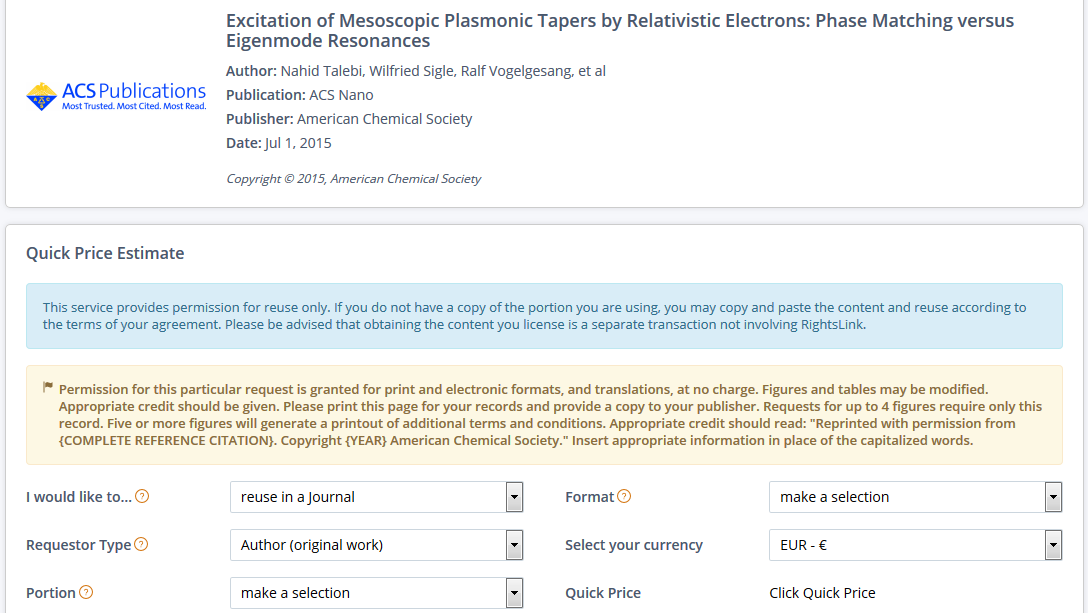

Supplement: Figures Licenses [file rsta20190599supp1.zip › Ref_33_license_a.PNG]

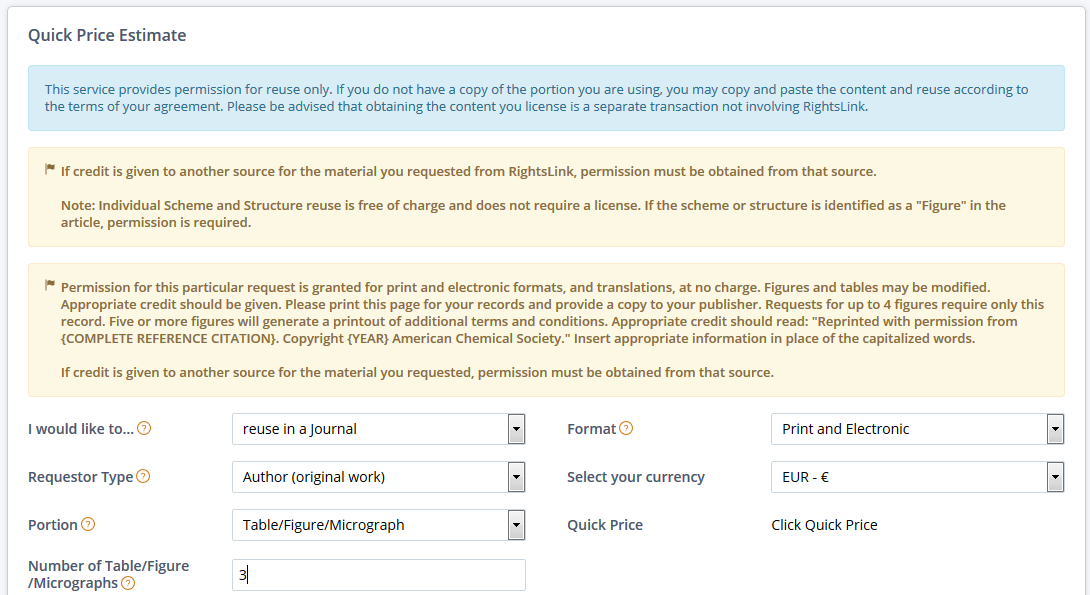

Supplement: Figures Licenses [file rsta20190599supp1.zip › Ref_33_license_b.PNG]

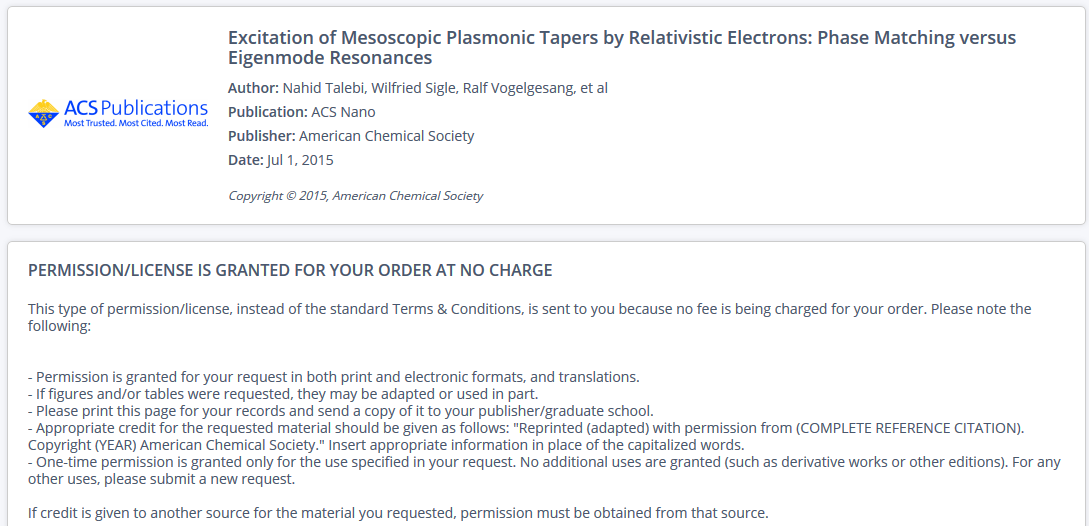

Supplement: Figures Licenses [file rsta20190599supp1.zip › Ref_33_license_c.PNG]

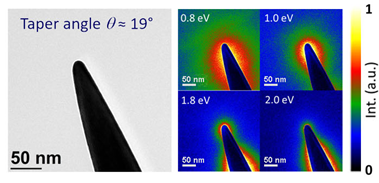

Supplement: Figures Licenses [file rsta20190599supp1.zip › Ref_47_a.PNG]

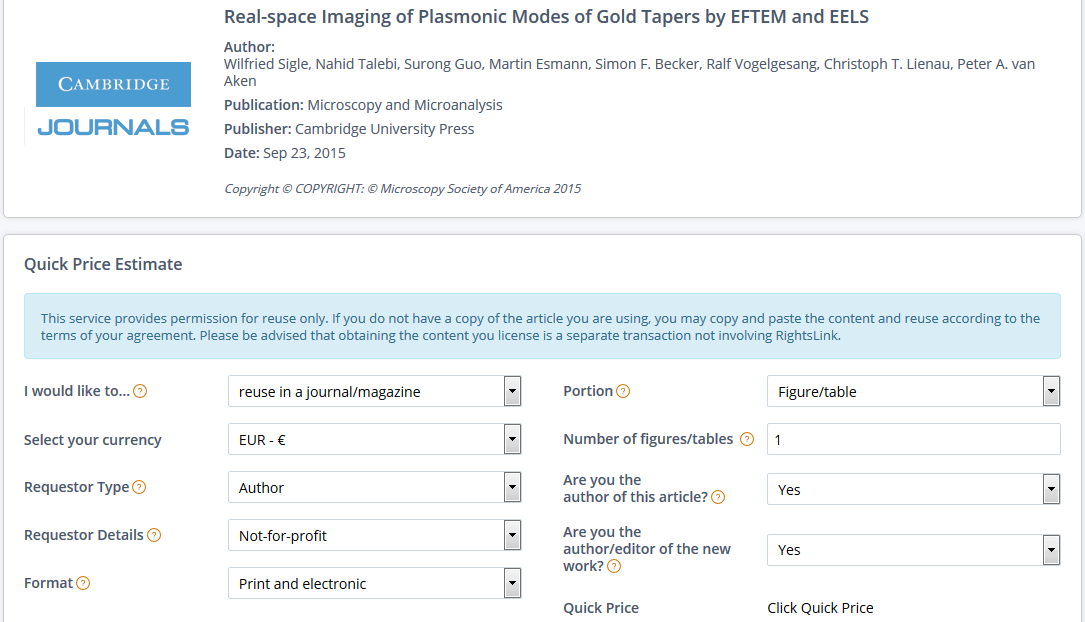

Supplement: Figures Licenses [file rsta20190599supp1.zip › Ref_47_license_a.PNG]

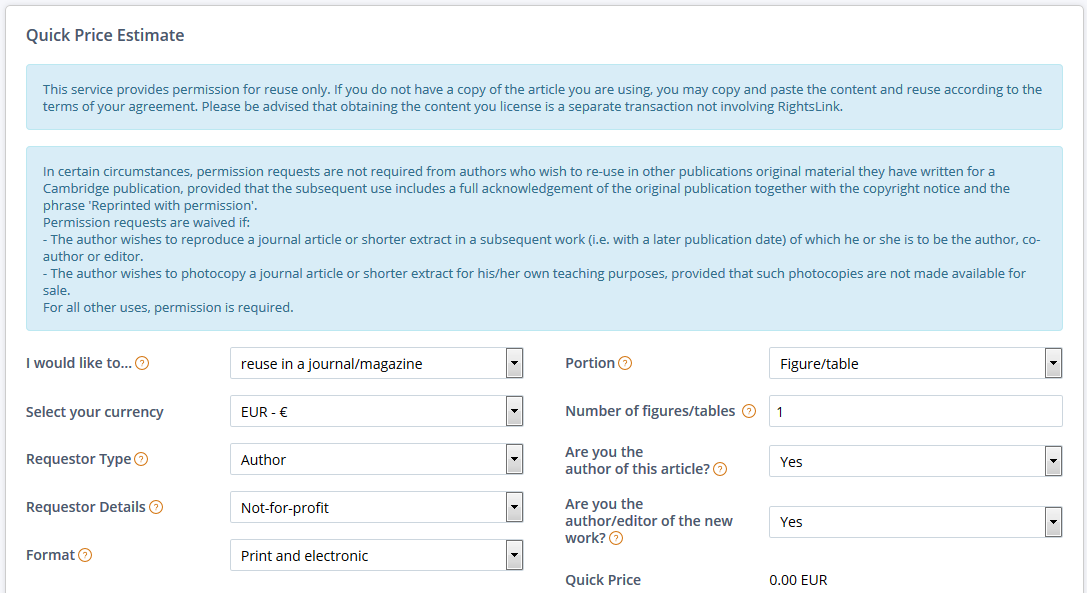

Supplement: Figures Licenses [file rsta20190599supp1.zip › Ref_47_license_b.PNG]

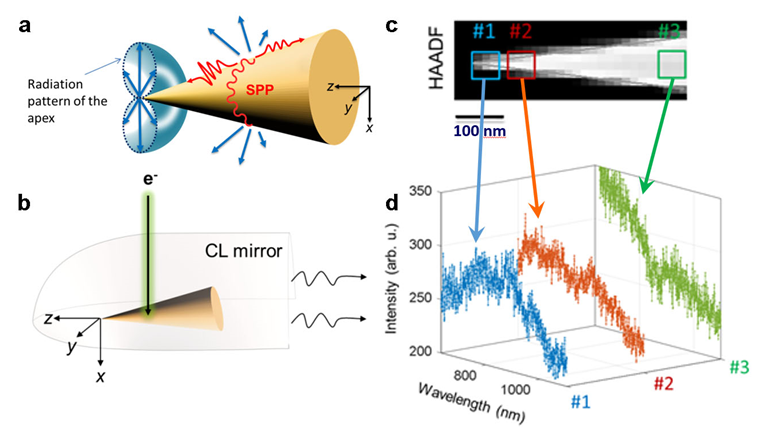

Supplement: Figures Licenses [file rsta20190599supp1.zip › Ref_66_a.PNG]

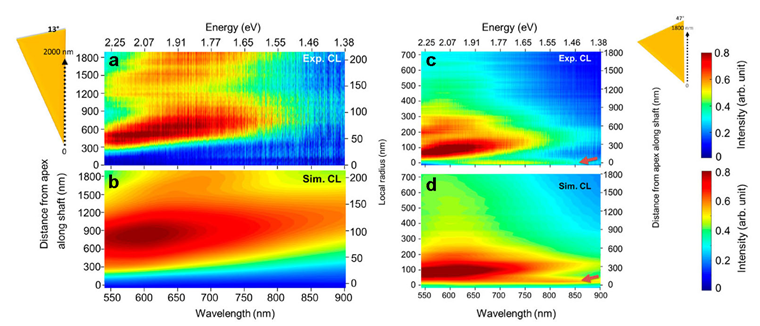

Supplement: Figures Licenses [file rsta20190599supp1.zip › Ref_66_b.PNG]

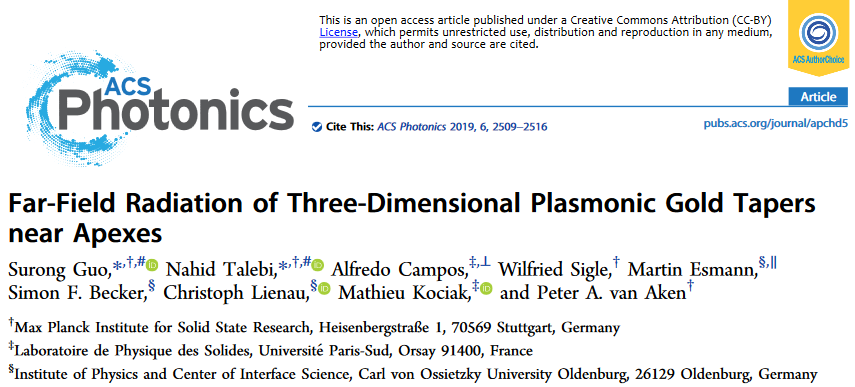

Supplement: Figures Licenses [file rsta20190599supp1.zip › Ref_66_CC-BY.PNG]

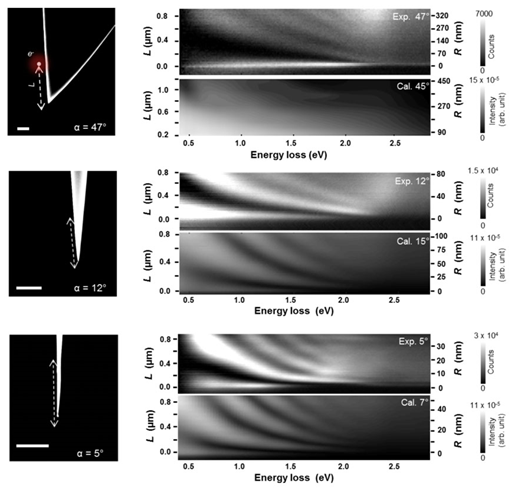

Supplement: Figures Licenses [file rsta20190599supp1.zip › Ref_82_a.PNG]

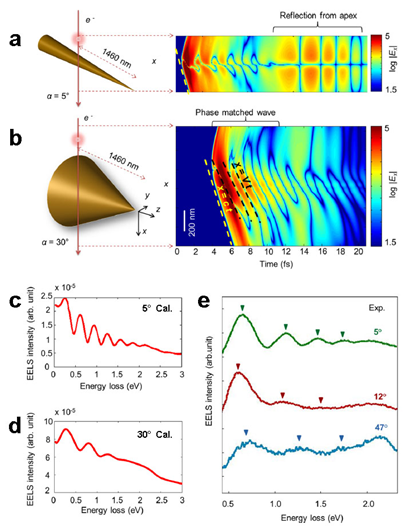

Supplement: Figures Licenses [file rsta20190599supp1.zip › Ref_82_b.PNG]

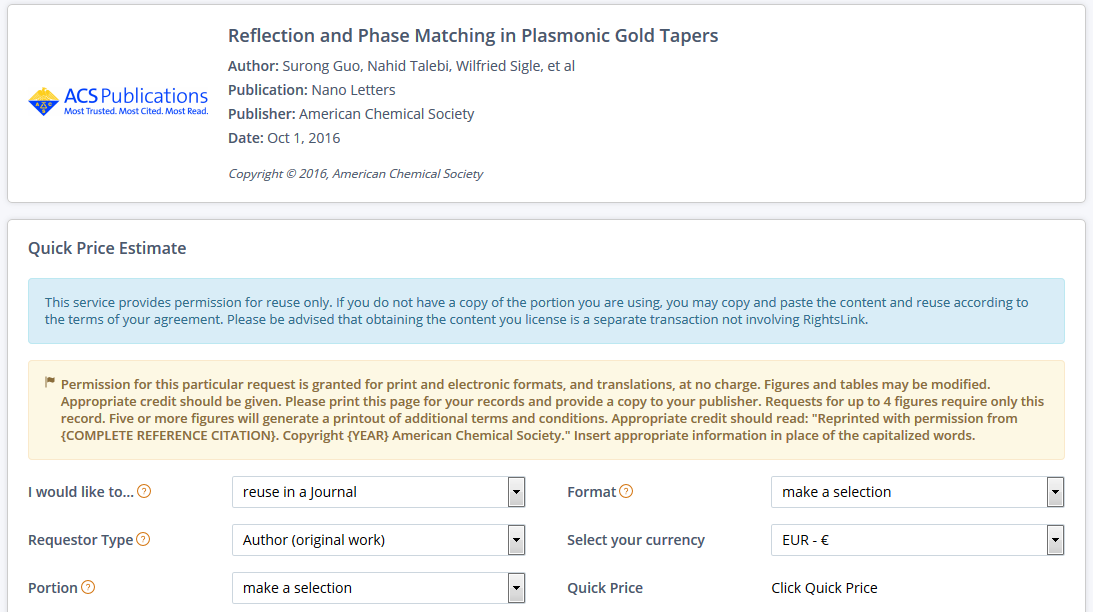

Supplement: Figures Licenses [file rsta20190599supp1.zip › Ref_82_license_a.PNG]

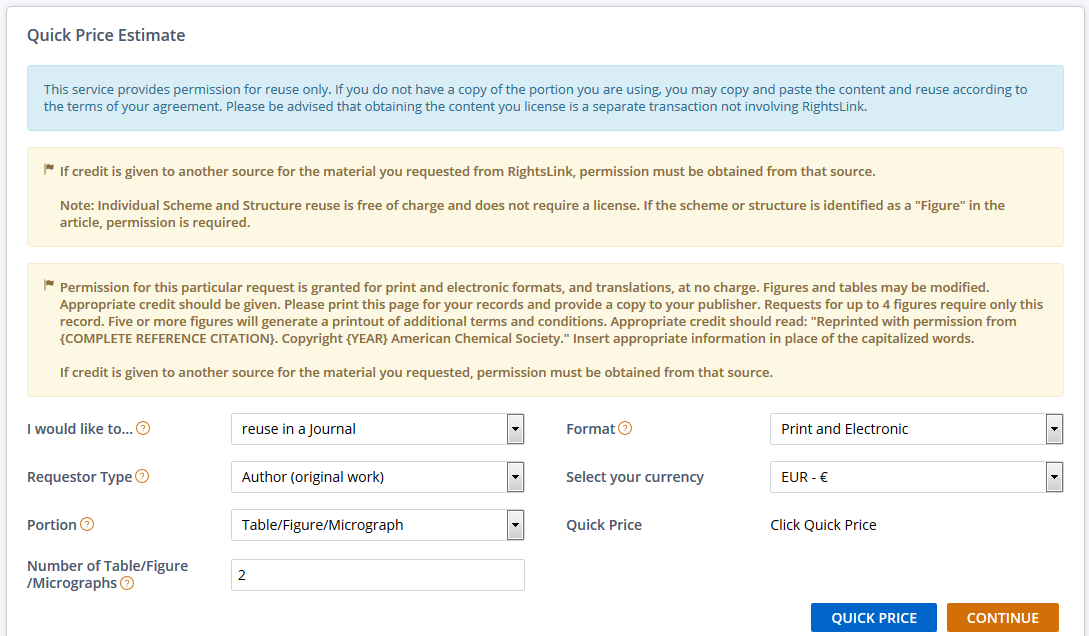

Supplement: Figures Licenses [file rsta20190599supp1.zip › Ref_82_license_b.PNG]

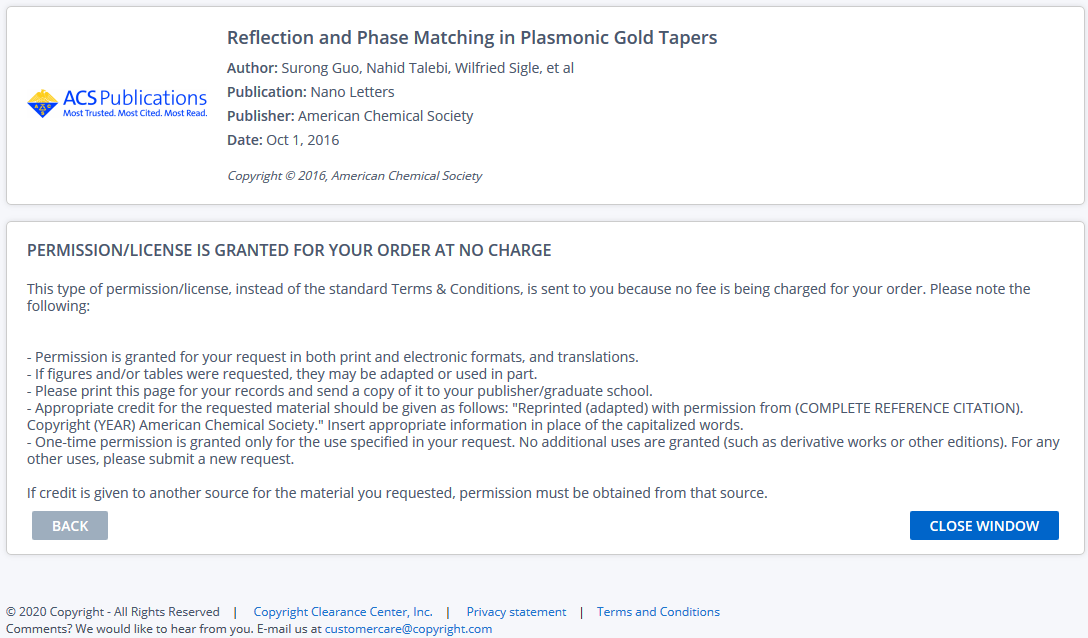

Supplement: Figures Licenses [file rsta20190599supp1.zip › Ref_82_license_c.PNG]

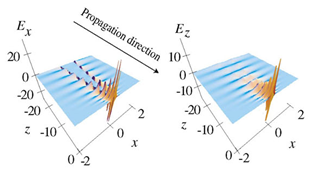

Supplement: Figures Licenses [file rsta20190599supp1.zip › Ref_5.PNG]
