# Supplementary material for: Probing plasmonic excitation mechanisms and far-field radiation of single-crystalline gold tapers with electrons
Source: Philos Trans A Math Phys Eng Sci. 2020 Oct 26;378(2186):20190599. doi: 10.1098/rsta.2019.0599 (PMC7661279; doi:10.1098/rsta.2019.0599)
Supplement: Ref 5 license author [file rsta20190599supp2.pdf]

# American Physical Society
# Reuse and Permissions License

27-Mar-2020

This license agreement between the American Physical Society ("APS") and Robin Lingstädt ("You") consists of your license details and the terms and conditions provided by the American Physical Society and SciPris.

## Licensed Content Information

| | |
|---|---|
| **License Number:** | **RNP/20/MAR/024124** |
| **License date:** | 27-Mar-2020 |
| **DOI:** | 10.1103/PhysRevLett.93.137404 |
| **Title:** | Nanofocusing of Optical Energy in Tapered Plasmonic Waveguides |
| **Author:** | Mark I. Stockman |
| **Publication:** | Physical Review Letters |
| **Publisher:** | American Physical Society |
| **Cost:** | USD $ 0.00 |

## Request Details

**Does your reuse require significant modifications**: No

| | |
|---|---|
| **Specify intended distribution locations:** | Worldwide |
| **Reuse Category:** | Reuse in a journal/magazine |
| **Requestor Type:** | Academic Institution |
| **Items for Reuse:** | Figures/Tables |
| **Number of Figure/Tables:** | 1 |
| **Figure/Tables Details:** | Figure 3 |
| **Format for Reuse:** | Print and Electronic |
| **Total number of print copies:** | More Than 10000 |

## Information about New Publication:

| | |
|---|---|
| **Publisher:** | The Royal Society |
| **Publication:** | Philosophical Transactions A |
| **Publication Date:** | Apr. 2020 |
| **Article Title:** | Probing plasmonic excitation mechanisms and far-field radiation of single-crystalline gold tapers with electrons |
| **Author(s):** | Robin Lingstädt, Nahid Talebi, Surong Guo, Wilfried Sigle, Alfredo Campos, Mathieu Kociak, Martin Esmann, Simon F. Becker, Eiji Okunishi, Masaki Mukai, Christoph Lienau and Peter A. van Aken |

## License Requestor Information

| | |
|---|---|
| **Name:** | Robin Lingstädt |
| **Affiliation:** | Individual |
| **Email Id:** | r.lingstaedt@fkf.mpg.de |
| **Country:** | Germany |

# American Physical Society
# Reuse and Permissions License

**TERMS AND CONDITIONS**

The American Physical Society (APS) is pleased to grant the Requestor of this license a non-exclusive, non-transferable permission, limited to Print and Electronic format, provided all criteria outlined below are followed.

1. You must also obtain permission from at least one of the lead authors for each separate work, if you haven't done so already. The author's name and affiliation can be found on the first page of the published Article.

2. For electronic format permissions, Requestor agrees to provide a hyperlink from the reprinted APS material using the source material's DOI on the web page where the work appears. The hyperlink should use the standard DOI resolution URL, http://dx.doi.org/{DOI}. The hyperlink may be embedded in the copyright credit line.

3. For print format permissions, Requestor agrees to print the required copyright credit line on the first page where the material appears: "Reprinted (abstract/excerpt/figure) with permission from [(FULL REFERENCE CITATION) as follows: Author's Names, APS Journal Title, Volume Number, Page Number and Year of Publication.] Copyright (YEAR) by the American Physical Society."

4. Permission granted in this license is for a one-time use and does not include permission for any future editions, updates, databases, formats or other matters. Permission must be sought for any additional use.

5. Use of the material does not and must not imply any endorsement by APS.

6. APS does not imply, purport or intend to grant permission to reuse materials to which it does not hold copyright. It is the requestor's sole responsibility to ensure the licensed material is original to APS and does not contain the copyright of another entity, and that the copyright notice of the figure, photograph, cover or table does not indicate it was reprinted by APS with permission from another source.

7. The permission granted herein is personal to the Requestor for the use specified and is not transferable or assignable without express written permission of APS. This license may not be amended except in writing by APS.

8. You may not alter, edit or modify the material in any manner.

9. You may translate the materials only when translation rights have been granted.

10. APS is not responsible for any errors or omissions due to translation.

11. You may not use the material for promotional, sales, advertising or marketing purposes.

12. The foregoing license shall not take effect unless and until APS or its agent, Aptara, receives payment in full in accordance with Aptara Billing and Payment Terms and Conditions, which are incorporated herein by reference.

13. Should the terms of this license be violated at any time, APS or Aptara may revoke the license with no refund to you and seek relief to the fullest extent of the laws of the USA. Official written notice will be made using the contact information provided with the permission request. Failure to receive such notice will not nullify revocation of the permission.

14. APS reserves all rights not specifically granted herein.

15. This document, including the Aptara Billing and Payment Terms and Conditions, shall be the entire agreement between the parties relating to the subject matter hereof.

**Betreff:** Re: permission to reuse figure for review paper
**Von:** Mark I Stockman <mstockman@gsu.edu>
**Datum:** 3/28/2020, 9:06 PM
**An:** Robin Lingstädt <r.lingstaedt@fkf.mpg.de>
**Kopie (CC):** Mark I Stockman <mstockman@gsu.edu>

The permission to reuse the figure is granted.

Best regards,

Mark Stockman

On 3/27/2020 6:45 AM, Robin Lingstädt wrote:

> Dear Prof. Stockman,
>
> for our review paper about our recent work on plasmonic gold tapers,
> which will appear in the "Philosophical Transactions of the Royal Society"
> we would like to include figure 3 of your paper (Ref_5.PNG)
>
> > Nanofocusing of Optical Energy in Tapered Plasmonic Waveguides
> > Phys. Rev. Lett. 93, 137404 – Published 23 September 2004
> > https://doi.org/10.1103/PhysRevLett.93.137404
>
> I received the "Reuse and Permissions License" from the American Physical Society (Ref_5_license.pdf),
> but according to their terms and conditions I need to get your permission as well.
>
> I am very sorry to bother you with such a small issue.
>
> With best regards from Stuttgart, Germany,
> Robin Lingstädt

Mark

______________________________
Mark I. Stockman, PhD, DSc
Regents' Professor of Physics
GSU Center for Nano-Optics (CeNO), Director

Department of Physics and Astronomy
Georgia State University (GSU), Atlanta, GA, USA

Phone: +1-678-457-4739 (worldwide mobile)
Fax: 404-413-6025 (limited use; PDF by e-mail preferred)
Web site: http://www.phy-astr.gsu.edu/stockman
